# Quality of Life and Societal Cost in Autistic Children: An Exploratory Comparative Study Pre- and Post-Diagnosis

**DOI:** 10.1007/s10803-025-06760-9

**Published:** 2025-03-09

**Authors:** Leontine W. ten Hoopen, Pieter F. A. de Nijs, Kirstin Greaves-Lord, Manon H. J. Hillegers, Werner B. F. Brouwer, Leona Hakkaart-van Roijen

**Affiliations:** 1 Department of Child and Adolescent Psychiatry/Psychology, Erasmus MC, Sophia Children’s Hospital, P.O. Box 2040, 3000 CA Rotterdam, The Netherlands; 2 Erasmus School of Health Policy & Management, Erasmus University Rotterdam, Burgemeester Oudlaan 50, 3062 PA Rotterdam, The Netherlands; 3 Department of (Youth) Mental Health and Autism, Jonx, Autism Team Northern-Netherlands, Lentis Psychiatric Institute, Groningen, The Netherlands; 4 Department of Psychology, Clinical Psychology and Experimental Psychopathology Unit, University of Groningen, Groningen, The Netherlands

**Keywords:** Autistic disorder, Child, Cost and cost analysis, Burden of illness, Diagnosis, Health services, Quality of life, Societal cost

## Abstract

Previous studies showed an impact of autism on the child’s quality of life and societal costs, although little is known about changes from pre- to post-diagnosis. Therefore, our study explored the utilization of health-related services and associated costs in a group of 36 clinically referred Dutch children with autism (aged 2–10 years), pre- and post-diagnosis. Taking a broad societal perspective, we included the child’s quality of life, educational needs, and absenteeism in school and leisure activities. Furthermore, we assessed the service utilization in various categories, such as healthcare, youth care, and school guidance. The caregivers, mainly the parents, provided the information. Special needs daycare was the overall major cost driver pre- and post-diagnosis. We found a non-significant decrease in total annual costs (from €6513 to €5060). Post-diagnosis, healthcare costs were halved, changing towards less somatic and more mental healthcare. No shift to adjacent cost categories was seen. At the same time, the child’s quality of life improved not significantly from 0.58 to 0.66 (EQ-5D-3L), with significantly fewer everyday activity problems. In addition, we observed a trend of less school absenteeism after the diagnosis. Regression analysis identified pre-diagnostic costs as the sole independent factor influencing post-diagnostic costs, explaining 23% of the variance. Our findings suggest the importance of timely clarification of autism as a foundation for informed intervention and treatment planning. This could potentially result in improved quality of life, appropriate service allocation, and reduced societal costs.

**Supplementary Information:**

The online version contains supplementary material available at 10.1007/s10803-025-06760-9.

## Introduction

Autism is a neurodevelopmental condition often manifesting in early childhood with challenges in social responsiveness and communication, as well as specific sensory, restricted, and repetitive behavior (American Psychiatric Association [APA], 2013; Lord et al., 2022). There is a variety of autism trait levels, experienced problems, and personal strengths among children and within children over time (Lord et al., 2022; Volkmar et al., 2014). Frequently, children with autism have co-occurring emotional, behavioral, and somatic problems (Lai et al., 2019; Muskens et al., 2017). Nowadays, circa 1 to 2% of children worldwide are diagnosed with autism (Lord et al., 2022; Zeidan et al., 2022). While the diagnostic category of the *Diagnostic and Statistical Manual of Mental Disorders* (*DSM*) is “Autism Spectrum Disorder”, we use the terms “autism” and “autistic” to meet community preferences (Bottema-Beutel et al., 2021).

Autism and its co-occurring problems may have a broad impact on children, associated with impaired quality of life (Kuhlthau et al., 2013; Ten Hoopen et al., 2020), more school absenteeism (Munkhaugen et al., 2017) and more special educational needs (Lavelle et al., 2014) than in typically developing peers. Autistic children often need lifelong support and a broad range of services with an early start of intervening to maximize outcomes (Lord et al., 2022; Volkmar et al., 2014). As a consequence, higher service utilization and associated costs for autistic children than non-autistic children, with more healthcare, educational, and social service needs, have been found (Rogge & Janssen, 2019; Zuvekas et al., 2021).

For appropriately allocating services and understanding actual societal costs, information on the impact of and the needed services for autistic children is essential (Lord et al., 2022). However, service utilization and cost estimates vary substantially across studies and countries because of different resources and financing, healthcare systems, data sources, and sample definitions (Rogge & Janssen, 2019). For example, many studies focus on medical expenses for autistic children using insurance claim datasets, primarily in the United States (Flanders et al., 2007; Leslie & Martin, 2007; Liptak et al., 2006; Mandell et al., 2006; Shimabukuro et al., 2008). As a result, these studies are limited in their representativeness of children with an autism diagnosis in their patient files who use medical healthcare services reimbursed by specific health insurance programs. Other, mainly European, studies are bottom-up, real-life cost calculations in diagnostically well-defined groups based on self- or caregiver reports on broad societal service utilization and costs (Barrett et al., 2012; Höfer et al., 2022; Järbrink, 2007; Knapp et al., 2009). Another source of variability is the included service and cost categories. In some studies, educational and community services are the major cost drivers, not medical services (Järbrink, 2007; Knapp et al., 2009). Lavelle et al. (2014) found caregiver time to account for many of the total costs in a US study, including medical, educational, and therapy services. Buescher et al. (2014) reported special education services and caregiver productivity loss as major cost drivers in a US and UK study with medical and non-medical costs, including residential care.

Identified factors associated with service utilization and costs in autistic children are symptom severity (Barrett et al., 2012), co-occurring conditions (Höfer et al., 2022; Peacock et al., 2012), and age (Barrett et al., 2012; Cidav et al., 2013). Barrett et al. (2012) found higher costs with more symptom severity in 152 two-to-five-year-old autistic children in the United Kingdom. Peacock et al. (2012) showed more costs with co-occurring developmental disorders, intellectual disability, and seizure disorders in 8398 autistic children aged 1–17 years in the United States. Cidav et al. (2013) found higher age associated with more psychiatric medication, (semi-)institutional care, and respite services but less community care in a United States study of 94,201 autistic, Medicaid-enrolled children aged 3–20 years.

Interestingly, Shimabukuro et al. (2008) showed higher healthcare utilization and costs in a privately insured group of autistic children in the United States in the year of the medical diagnosis. The impact of receiving a diagnosis on the needs, utilized services, and associated costs of autistic children is still an understudied topic. Flanders et al. (2007) revealed markedly higher healthcare costs in the six months preceding the diagnosis compared to the year following it in the Medicaid data from the United States of 731 autistic children. The elevated pre-diagnostic healthcare costs might be due to the complex process toward an autism diagnosis, often ruling out other potential diagnoses (Flanders et al., 2007; Lord et al., 2022; Volkmar et al., 2014). Early clarification of autism with proactive intervention planning has been emphasized (Hyman et al., 2020). However, a comprehensive understanding of the health-related service utilization and societal costs associated with autism in children pre- and post-diagnosis is still lacking.

Hence, we examined the health-related medical and non-medical service utilization and the associated costs *before* and *after* an autism diagnosis in clinically referred children based on real-life data. According to guidelines for evaluating health-related service utilization and associated costs, a societal perspective is recommended (Hakkaart et al., 2024). The societal perspective includes as many relevant actors, services, and costs as possible, regardless of the bearer of the costs. Therefore, the services in our study included the healthcare sector (with somatic and mental healthcare), the child and family sector (for example, personal homework guidance), and other sectors (such as school guidance and youth care). With the broader societal perspective to assess the burden of illness, we included the child’s quality of life, educational needs, and school and leisure activity attendance. We aimed to identify improved quality of life, reduced school and leisure activity absenteeism, and decreased health-related service utilization and costs after the diagnosis. Additionally, we examined the factors affecting post-diagnostic health-related service costs. Our objective was to gain insight into the quality of life, actual needed services, and broad societal costs for autistic children before and after receiving the diagnosis, ultimately improving the quality of life and ensuring the appropriate allocation of services.

## Methods

### Data Collection

We collected data within the Social Spectrum Study, a Dutch multicenter study of child, family, and societal factors associated with autism traits in clinically referred children (Duvekot et al., 2017). The study involved 134 autistic children aged 2–10, referred to six participating child and adolescent mental health (CAMH) centers for various developmental, emotional, and behavioral reasons such as anxiety, autistic features, aggressive behavior, or learning problems, and selected with the Autism Diagnostic Observation Schedule, Second Edition (ADOS-2; Lord et al., 2012).

Information on the utilization of health-related services before (T0) and after diagnosis (T2) was provided by the primary caregiver (97% biological parents). At the time of referral, caregivers filled out this questionnaire for the first time to receive information about pre-diagnostic service utilization (T0). After receiving the information, the diagnostic procedure would take place. Approximately one year after the diagnostic procedure, caregivers were approached again to complete the questionnaire (T2). Data on T0 was available for 74 out of 134 autistic children (55%); the autistic children with questionnaires were, on average, slightly younger, more often boys, more of Dutch origin, living with both biological parents, and had slightly more autism traits and higher total problem scores, than the autistic children without questionnaires, although all differences were not statistically significant. At T2, caregivers filled out the questionnaire for the second time for 36 of the 74 autistic children with a questionnaire at T0 (49%), with an average interval of 23.39 months (*SD* = 3.75). No children were excluded from the study. We found that children with available questionnaires (*n* = 36) were, on average, slightly younger, more often boys, more likely to be of Dutch origin, living with both biological parents, presented with a higher level of autism traits and higher total problem scores, compared to children without both questionnaires (*n* = 98), although all not statistically significant.

Caregivers were also asked twice to complete questionnaires about the child’s general characteristics, emotional and behavioral problems, educational needs, school and leisure activity attendance, and quality of life. The child’s IQ was obtained from the patient file or assessed by a trained psychologist (Duvekot et al., 2017). Additionally, caregivers provided reports on their own emotional and behavioral problems, parenting stress, and family problems.

The study, as well as the information and consent/assent procedure, were approved by the medical ethics committee of the Erasmus Medical Center and the participating CAMH centers prior to the data collection (MEC-2011-078), and the caregivers provided written consent for all the assessments.

### Study Population

For this study, children with an ADOS-2 classification of ‘Autism’ or ‘Autism Spectrum Disorder’ were selected. Also, we calculated the ADOS calibrated severity score (CSS; Gotham et al., 2009), with higher scores reflecting more autism traits during the observation. The ADOS has good psychometric properties (Lord et al., 2012). The child’s emotional and behavioral problems were scored on the Child Behavior Checklist (CBCL; Achenbach & Rescorla, 2000, 2001) by the caregivers, with higher scores for more problems. The CBCL 1.5–5 was used for children up to six years (99 items, 42% at T0), and the CBCL 6–18 (118 items, 58% at T0) for the older children. We used total *T* scores because of the different total item numbers of both versions. The versions have good psychometric properties (Achenbach & Rescorla, 2000, 2001).

Caregivers used the Adult Self Report (ASR; Achenbach and Rescorla et al., 2003) to assess their own emotional and behavioral problems (total score range 0–240), the Parenting Stress Questionnaire (OBVL; Vermulst et al., 2015) for their parenting stress (total score range 34–136), and the General Functioning Scale of the McMasters Family Assessment (FAD; Epstein et al., 1983) for family problems (total score range 12–48). Higher scores indicated more problems.

### Health-Related Quality of Life

The caregiver assessed the child’s quality of life (QoL) with the proxy version of the EQ-5D-3L (The EuroQoL Group, 1990; www.euroqol.org). The five domains, ‘*mobility*’, ‘*self-care*’, ‘*usual activities*’, ‘*pain/discomfort*’, and ‘*anxiety/depression*’, were rated with three-level answers (0 = no problems, 1 = some or moderate problems, and 2 = a lot of problems). The EQ-5D-3L utility score was calculated by using the Dutch set of the general population, not autism-specific, preference values (Lamers et al., 2006), anchored on 0 (*state equal to being dead*) and 1 (*state of perfect health*). Negative utility scores are possible (i.e., ‘*states worse than being dead’, involving combinations of severe problems in several health domains, like pain and depression*).

### Health-Related Service Utilization and Associated Costs

The parent form of the Treatment Inventory of Costs in Patients with Mental Health Problems for Children (TiC-P-Youth or TiC-P-Y, www.imta.nl; Bouwmans et al., 2013; Hakkaart et al., 2015) was completed by caregivers pre- and post-diagnosis. The initial data collection was done around the time of the referral and before the first appointment or intake at the CAMH to avoid measuring service utilization related to the diagnostic assessment. From a societal perspective, according to cost measurement guidelines (Hakkaart et al., 2024), we included twenty-nine potentially utilized health-related services in three cost categories, regardless of whether the costs were related to autism and who incurred them (Supplementary File S1). Using the TiC-P-Y, caregivers reported the frequency and number of utilized services (e.g., contacts, consultations, admission days) for the child in the cost categories. The category ‘*healthcare sector costs*’ encompassed fifteen (para)medical consultations, out- and inpatient services, and prescribed and over-the-counter medication use for somatic and mental healthcare. The category ‘*child and family costs*’ covered two out-of-pocket payments for homework guidance and diets. The category ‘*other sector costs*’ related to twelve services, such as youth care services, extra school guidance, special needs (after-school) daycare, (weekend) foster care, community crisis care, and police contacts. As a standard part of the TiC-P-Y, the caregivers assessed the child’s health-related absenteeism in school and leisure activities. Information about the caregiver’s informal care and productivity loss was not collected at both time points because of the focus on child service utilization and balancing the caregiver’s administrative load in the Social Spectrum Study, although recommended (Hakkaart et al., 2024) and possible with a module of the TiC-P-Y. The recall period was 3 months in the trade-off between a representative and accurate recall.

We calculated annual service utilization costs using the bottom-up methodology by multiplying the reported volume of services and medication with unit prices. We used the reference unit prices from the Dutch Costing Manual (Hakkaart et al., 2015) indexed to 2021 euro values (Supplementary Files S2 and S3). For unavailable reference unit prices, we used public sources such as professional associations, the National Budget Information Institute (NIBUD; www.nibud.nl), and the Dutch government (www.rijksoverheid.nl). Medication unit prices were obtained via the National Health Care Institute (Zorginstituut Nederland). For over-the-counter medication, average market prices were derived from consumer websites (Supplementary File S4 and S5).

### Statistical Analyses

The mean and standard deviation of total annual costs for each service and medication utilized and for each cost category (i.e., healthcare sector costs, child and family costs, and other sector costs) were calculated. At the most, reports of three users per service were missing (e.g., specialized daycare, remedial teaching, foster care), with a mean missing of 4%. We imputed missing values using multiple imputation with 30 iterations for all analyses of service utilization and associated costs. The imputation equation included the child’s age, autism severity, and quality of life as covariates.

Changes in the child’s quality of life, school and leisure activity absenteeism, and service utilization with associated costs over time were examined with the Wilcoxon signed-rank test or McNemar test. Testing the influence of the child’s age and autism traits on the quality of life and the service utilization costs, we performed univariate single-variable regression models at both time points. To explore potential associations of post-diagnostic health-related service costs, we tested univariate single-variable regression models with pre-diagnostic health-related service costs and child and caregiver variables. Next, we included the significantly associated variables in a multivariable regression analysis with post-diagnostic health-related service costs as the dependent variable.

Because of the study's exploratory nature, we considered a p-value of < 0.05 significant.

## Results

### Study Population

Table 1 presents the characteristics of the study population. Most children were boys (81%), lived with both biological parents (83%), and were of Dutch origin (86%). Before diagnosis, the mean age was 6.14 years (*SD* = 2.51, *Mdn* = 6.50*, IQR* 3.25–8.00), the mean full-scale IQ was 92.65 (*SD* = 17.14, *Mdn* = 94.50, *IQR* 80.75–112.00), and the mean CBCL total problem *T* score was 69.54 (*SD* = 8.23, *Mdn* = 71.00, *IQR* 65.00–74.00) with a clinical score (≥ 70) in 57% of the children. On the ADOS, the mean CSS was 6.56 (*SD* = 1.96, *Mdn* = 6.00, *IQR* 5–8), with 67% of the children having an ADOS classification of ‘*Autism*’ (*n* = 24) versus 33% with an ADOS classification of ‘*Autism Spectrum Disorder*’ (*n* = 12). Post-diagnosis, the mean CBCL total problem T score of 66.72 (SD = 7.47, *Mdn* = 68.00, *IQR* 61.00–72.50), with a clinical score in 44% of the children, was significantly lower.

**Table 1:** Study population characteristics (*n* = 36)

	Characteristics	*M (SD)*/*n (%)*
Children	Sex (% boys)	29 (81%)
	*Age (years)*	
	T0	6.14 (2.51)
	T2	8.14 (2.60)
	Full-scale IQ	92.65 (17.14)
	Origin (% Dutch)^a^	31 (86%)
	*Living with both biological parents*	
	T0	30 (83%)
	T2	30 (83%)
	School attendance^b^	
	T1	31 (89%)
	T2	35 (97%)**
	*Learning problems*^b^	
	T1	19 (61%)^c^
	T2	17 (49%)* ^c^
	*Remedial teaching*^b^	
	T1	12 (39%)^c^
	T2	12 (34%)^c^
	ADOS-2 CSS	6.56 (1.96)
	*CBCL total problem T score*	
	T0	69.54 (8.23)
	T2	66.72 (7.47)*
	*CBCL total problem T score ≥ 70*	
	T0	20 (57%)
	T2	16 (44%)
	*EQ-5D-3L utility score*^b^	
	T1	0.58 (0.26)
	T2	0.66 (0.18)
Caregivers and family	Sex (% female)	34 (94%)
	*Age (years)*	
	T0	35.80 (5.02)
	T2	37.89 (5.13)
	Biological parent	35 (97%)
	Number of children in the family	2.42 (1.00)
	*Education*	
	Lower	8 (22%)
	Medium	18 (50%)
	Higher	10 (28%)
	Employed	26 (74%)
	ASR total problem score^b^	40.20 (25.17)
	OBVL total score^b^	64.26 (13.09)
	FAD total score^b^	22.39 (4.64)

*T0* pre-diagnostic phase *T1* diagnostic phase *T2* post-diagnostic phase. *p ≤ 0.05; **p ≤ *0.01*

*IQ* Intelligence Quotient *ADOS-2 CSS* Autism Diagnostic Observation Schedule Second Edition, Calibrated Severity Score *CBCL* Child Behavior Checklist *EQ-5D-3L* EuroQoL Five Domain Health Questionnaire *ASR* Adult Self Report *OBVL* Parenting Stress Questionnaire, *FAD* Family Assessment Device

^a^Origin was classified as ‘Dutch’ if both parents were born in the Netherlands

^b^First assessment at diagnostic phase (T1)

^c^Percentage of children attending school

### Health-Related Quality of Life

**Fig. 1:**
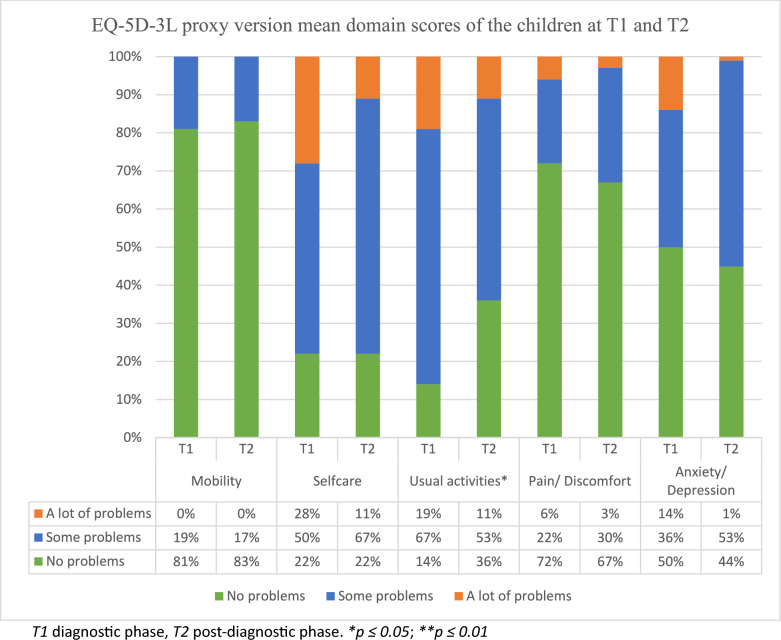
Distribution of mean problem domain score frequencies (%) of the children at the diagnostic phase (T1) and post-diagnostic phase (T2) on the EQ-5D-3L proxy version

Figure 1 shows the distribution of the EQ-5D-3L proxy reports of the children with frequencies (%) of problem levels and the total utility score. The children had the most problems (problem score ≥ 1 per domain) in the ‘*self-care*’ domain (T1 and T2: 78%) and the ‘*usual activities*’ domain (T1: 86%; T2: 64%). There were fewer but still substantial problems in the ‘*anxiety/depression*’ domain (T1: 50%; T2: 56%). The reduction of ‘*usual activities*’ problems was the only significant change (*p* = 0.016), meaning that children had fewer problems with everyday activities post-diagnosis. The children’s health-related quality of life improved from 0.58 (*SD* = 0.26) during the diagnostic phase to 0.66 (*SD* = 0.18) post-diagnosis, although this increase was not statistically significant (*p* = 0.095). In the univariate regression analyses, we found no significant effects of the child’s age and autism severity on the quality of life at both time points.

### Health-Related School and Leisure Activity Absenteeism

In the diagnostic phase (T1), 86% of the children (age in years *M* = 7.00; *SD* = 2.63; range 3–11) attended school, of whom 35% had special needs education. Of the children attending school, 61% experienced learning problems, with 53% in regular education and 47% in special needs education. Among children with learning problems, 63% received remedial teaching, with 58% in regular education and 42% in special needs education. Other education types included regular daycare (6%) or special needs daycare (8%). Post-diagnosis (T2), almost all children attended school (97%; with 31% special needs education) besides special needs daycare (3%). Fewer school-attending children experienced learning problems (49%), with 41% in regular education and 59% in special needs education. More children with learning problems received remedial teaching (71%), with 25% in regular education and 75% in special needs education.

We found no significant differences between health-related school and leisure activity absenteeism of the children post-diagnosis. In the diagnostic phase, school absenteeism was present in 53% with a mean of 8 days per year (*SD* = 10.97; *Mdn* = 4.00; max. 30 days), and missed leisure activities in 22% with a mean of 2.86 times per year (*SD* = 6.18; *Mdn* = 0.00; max. 28 times). Post-diagnosis, we found a non-statistically-significant trend (*p* = 0.177) of less school absenteeism in children (49%) with a mean of 5.20 days per year (*SD* = 7.76; *Mdn* = 0.00; max. 32 days) and about the same missed leisure activities in 23% with on average 2.46 times a year (*SD* = 5.74; *Mdn* = 0.00; max. 24; *p* = 0.693).

### Health-Related Service Utilization

Table 2 shows annual health-related service utilization and associated costs, including the percentage of children in the sample who utilized this service. Before the diagnosis (T0), the most commonly used services were general practitioner visits (58%), hospital outpatient care (39%), speech therapy (36%), school guidance (28%), remedial teaching (25%), and other services (28%, for example, personal home guidance). The most frequently used care included special needs daycare (176 days), community crisis care (112 days), and physiotherapy consultations (100). After the diagnosis (T2), we observed a change in service utilization. The most commonly used services were general practitioner visits (31%), mental healthcare (28%), speech therapy (22%), and remedial teaching (19%). Additionally, foster care (360 days) and special needs daycare (240 days) were most frequently used. Major changes over time (T0 to T2) were fewer general practitioner consultations (∆27%), fewer hospital outpatient clinic visits (∆25%), less guidance at school (∆22%), less speech therapy (∆14%), and fewer physiotherapy consultations (∆11%), more private psychological, psychotherapist, or psychiatric care (∆19%), and more mental healthcare (∆11%). Use of special needs after-school care, weekend foster care, police contacts, or detention were not reported at all. Although we found no change in total healthcare service utilization, there was a major increase in youth mental healthcare (public and private access) and a major decrease in somatic healthcare over time (with cost consequences in the next section).

**Table 2:** Total annual health-related service use and costs (€) of autistic children pre- and post-diagnosis (*n* = 36)

	T0 (pre-diagnosis)		T2 (post-diagnosis)		*P* costs
Health-related service	Respondents using the resource % (max. use; missing)	Mean costs (SD)	Respondents using the resource % (max. use; missing)	Mean costs (SD)	
General practitioner (consult)	**58.3 (20;0)**	168.42 (197.31)	**30.6 (16;0)**	78.05 (143.53)	0.033*
Physiotherapist (consult)	19.4 (100;0)	316.30 (899.28)	8.3 (48;0)	73.94 (317.17)	0.050*
Occupational therapist (consult)	5.6 (40;0)	61.62 (272.39)	0.0 (0;0)	0.00 (0.00)	0.180
Speech therapist (consult)	**36.1 (64;1)**	360.41 (623.94)	22.2 (96;0)	262.88 (656.82)	0.187
Dietician (consult)	5.6 (8;1)	8.01 (46.65)	2.7 (4;1)	3.89 (23.33)	0.655
Homeopath, acupuncturist (consult)	5.6 (4;1)	6.33 (36.65)	5.6 (16;0)	30.56 (150.16)	0.317
Social worker (consult)	8.3 (8;2)	16.46 (93.25)	5.6 (4;0)	15.56 (65.05)	1.000
Mental healthcare (consult)	16.7 (92;2)	452.33 (1950.19)	**27.8 (60;1)**	566.12 (1612.49)	0.106
Psychologist, psychiatrist—practice (consult)	0.0 (0;0)	0.00 (0.00)	19.4 (24;0)	186.22 (495.21)	0.017*
Youth care/welfare (contact)	19.4 (8;1)	81.17 (189.64)	11.1 (16;2)	61.25 (223.62)	0.391
Family guardian (contact)	11.1 (12;1)	47.11 (189.43)	5.6 (16;0)	71.11 (297.36)	0.180
Special needs daycare (day)	19.4 (176;3)	1,969.43 (7632.45)	5.6 (240;0)	2,321.00 (10,260.51)	0.892
Remedial teaching (contact)	25.0 (48;3)	141.41 (524.04)	19.4 (48;1)	231.11 (677.36)	0.832
Homework guidance—private (cost)	0.0 (0;2)	0.00 (0.00)	5.6 (1512;1)	82.00 (492.00)	0.317
Extra school guidance (contact)	27.8 (20;2)	40.51 (93.39)	5.6 (48;0)	35.62 (184.86)	0.205
Special needs after-school care (day)	0.0 (0;2)	0.00 (0.00)	0.0 (0;0)	0.00 (0.00)	1.000
Weekend foster care (day)	0.0 (0;2)	0.00 (0.00)	0.0 (0;0)	0.00 (0.00)	1.000
Community crisis care (day)	5.6(112;1)	774.16 (4515.49)	0.0 (0;0)	0.00 (0.00)	0.317
Foster care (day)	13.9 (48;3)	37.86 (163.10)	8.3 (360;0)	215.05 (1172.83)	0.285
Police (contact)	0.0 (0;1)	0.00 (0.00)	0.0 (0;0)	0.00 (0.00)	1.000
Detention (day)	0.0 (0;1)	0.00 (0.00)	0.0 (0;0)	0.00 (0.00)	1.000
Other services (contact)	27.8 (28;2)	262.19 (655.82)	16.7 (96;2)	429.42 (1467.25)	0.674
Hospital outpatient clinic (consult)	**38.9 (92;1)**	650.74 (1819.08)	13.9 (12;0)	89.49 (257.70)	0.006**
Hospital day admission (day)	5.6 (4;1)	22.64 (132.01)	2.8 (8;0)	44.02 (264.13)	0.655
Hospital admission (day)	5.6 (4;1)	159.14 (928.03)	0.0 (0;0)	0.00 (0.00)	0.317
Admission otherwise (day)	0.0 (84;1)	806.35 (4699.70)	0.0 (0;0)	0.00 (0.00)	0.317
Medication use, prescribed (day)	**44.4 (365;10)**	112.01 (386.90)	**52.8 (365;3)**	224.74 (417.47)	0.161
Medication use, over-the-counter (day)	11.1 (280;2)	3.78 (4.95)	**27.8 (365;3)**	10.38 (35.62)	1.000
Diet (use)	11.1 (0)	14.44 (61.15)	5.6 (0)	27.78 (166.67)	0.655
Total healthcare sector resources	94.4	3144.53 (6149.51)	83.3	1518.85 (1936.86)	0.201
Total child & family resources	5.6	14.44 (61.15)	5.6	109.78 (514.93)	0.285
Total other sector resources	66.7	3353.84 (8807.60)	41.7	3364.56 (11,305.44)	0.101
Total resource users and total costs	94.4	6512.81 (13,091.37)	86.1	5060.19 (12,092.33)	0.388

T0 pre-diagnostic phase, T2 post-diagnostic phase

Top 4 most utilized resources at the time point in bold; * p ≤ 0.05; ** p ≤ *0.01* (significant differences in bold)

From pre- to post-diagnosis, more children used prescribed medication (44 to 53%) and over-the-counter medication (11 to 28%). Pre-diagnosis, no psychoactive medication was prescribed, except anti-epileptic medication for one child and Melatonin for two children; post-diagnosis, caregivers reported the use of psychoactive medication for attention-deficit and hyperactivity disorder (ADHD) in thirteen children (Methylphenidate or Atomoxetine), anti-psychotic medication for behavioral regulation in six children (Aripiprazole, Risperidone, or Pipamperone), Melatonin in seven children, next to the same child still using anti-epileptic medication. The most used over-the-counter medication was Paracetamol at both times. Post-diagnosis, also Melatonin was twice reported in this category.

Although the number of total service users diminished by 8% over time with a reduction in ‘*healthcare’* service utilization by 11%, we saw a reduction in ‘*other sector*’ service utilization from pre-diagnosis (67%) to post-diagnosis (42%), meaning fewer children requiring community services such as community crisis care.

### Health-Related Service Costs

Table 2 shows the total annual post-diagnostic costs as lower than pre-diagnostic costs (€6513 at T0 versus €5060 at T2; *p* = 0.388). Of all three cost categories, the healthcare costs show the most remarkable fall over time (€3145 vs. €1519; *p* = 0.201), with significant decreases concerning the general practitioner (€168 vs. €78; *p* = 0.033), physiotherapy (€316 vs. €74; *p* = 0.050), and hospital outpatient clinic (€651 vs. €89; *p* = 0.006). In return, we found increased psychological, psychotherapeutic, or psychiatric consultation in small practices (€0 vs. €186; *p* = 0.017) and in mental healthcare (€452 vs. €566; *p* = 0.106). The major cost driver of the total ‘*healthcare sector*’ cost category shifted from admission otherwise pre-diagnosis to mental healthcare post-diagnosis. Within the ‘*other sector’* costs category, the primary shifts over time were a total reduction in community crisis care and an increase in foster care. Slightly more ‘*child and family*’ costs and ‘*other sector*’ costs were reported. Special needs daycare remained the major cost driver pre- and post-diagnosis overall.

Univariate regression analyses of the total costs and costs in the three cost categories (i.e., ‘*healthcare sector*’, ‘*child and family*’, and ‘*other sector*’ costs) showed no significant effect on the child’s age and autism level.

Pre- and post-diagnostic total costs were associated (*p* = 0.002), with pre-diagnostic costs explaining 23% of the variance of the post-diagnostic costs. Also, total costs and child quality of life seemed inversely related (*p* = 0.028) post-diagnosis, meaning better quality of life in the child with lower costs (Supplementary File 6). However, multivariable regression showed no significant associations between post-diagnostic total costs and child variables, such as age, autism severity, or emotional and behavioral problems, nor with caregiver problems, parenting stress, or family functioning in the study (Table 3).

**Table 3:** Multivariable regression analysis with total annual costs of autistic children at T2 as dependent variable (significant variables from univariate single variable regression analyses with *p* ≤ 0.05)

Variables	*B*	95% CI for B		*SE B*	*β*	*p*	*R*^*2*^	*Adj. R*^*2*^
		LB	UB					
Model						0.003	0.297	0.254**
Constant	12,155.899	−2367.818	26,679.616	7138.662				
*Child variables*								
Age	–	–	–	–	–	–		
Functioning (CBCL)	–	–	–	–	–	–		
Autism (ADOS-2)	–	–	–	–	–	–		
Quality of life at T1 (EQ-5D)	–	–	–	–	–	–		
Quality of life at T2 (EQ-5D)	−14,605.205	−34,791.428	5581.017	9921.883	−0.227	0.150		
Total annual costs at T0	0.395	0.105	0.685	0.143	0.427**	0.009		
*Caregiver variables*								
Age caregiver	–	–	–	–	−	–		
Functioning caregiver (ASR)	–	–	–	–	–	–		
Parenting stress (OBVL)	–	–	–	–	–	–		
Family functioning (FAD)	–	–	–	–	–	–		

*p ≤ 0.05; **p ≤ 0.01(significant results in bold)

*CI* confidence interval, *LB* Lower bound, *UB* Upper bound, *T0* Pre-diagnostic phase, *T1* Diagnostic phase*, T2* Post-diagnostic phase*, CBCL* Child Behavior Checklist, *ADOS-2* Autism Diagnostic Observation Schedule-2, *EQ-5D* EuroQoL Five Domain Health Questionnaire, *ASR* Adult Self-Report, *OBVL* Opvoedingsbelasting vragenlijst [Parenting Stress Questionnaire], *FAD* Family Assessment Device

## Discussion

This study is the first to explore the impact of autism in clinically referred children *before* and *after* receiving the diagnosis, from a broad societal perspective, with quality of life, educational needs, school and leisure activities, health-related service utilization, and associated costs. The total mean annual costs for a child diminished from €6513 to €5060 between these time points. The number of children utilizing health-related services decreased 8% during this period. We observed trends in the healthcare sector towards increased mental healthcare utilization and decreased somatic healthcare utilization, with over 50% reduction in mean total annual healthcare costs post-diagnosis (from €3145 to €1519). This change might reflect the consequences of more targeted care and support following an autism diagnosis. Moreover, the reduction of community crisis care and the increase in foster care over time could indicate more lasting and stable solutions for family crises. There was no substitution of care to other sector resource utilization post-diagnosis. At both time points, special needs daycare was the major cost driver. In the same period, the child’s quality of life seemed to improve from 0.58 to 0.66, with a significant decrease in problems with ‘usual activities’. Fewer caregivers reported learning problems than before, while more children received remedial teaching. We observed trends of less school absenteeism post-diagnosis. Most shifts in education type over time seemed to be due to the child's aging; the education type was relatively stable.

Although international comparison of service utilization and costs is difficult due to variations in cost categories and healthcare systems, we found our mean annual costs to be in the upper middle region (Croteau et al., 2017; Höfer et al., 2022; Rogge & Janssen, 2019). For example, Croteau et al. (2017) found mean annual costs of 11,009 to 6937 Canadian dollars in the first to the second year post-diagnosis in 1227 autistic children and adolescents to 25 years old, including psychiatric hospitalization, psychiatric medical visits, and the use of psychoactive medication, using population-based data; psychiatric hospitalization was the major cost driver. Although the mean number of psychiatric visits decreased over the years post-diagnosis, the use of psychoactive medication increased. In comparison, Höfer et al. (2022) showed mean annual costs of €4177 for autistic children to 11 years old visiting outpatient clinics in a German health service cost study, with occupational therapy, psychiatric inpatient, and autism outpatient care as major cost drivers. Finally, Barrett et al. (2012) reported mean annual costs of $9194 (corrected to 2018 US$ by Rogge & Janssen, 2019) in autistic two-to-five-year-old children in the UK with community health, social, and educational services, childcare, and hospital-based health services as major cost drivers. Possible explanations for differences with our study’s service utilization and cost estimates could be (a) broader categories of community services and out-of-pocket expenses in addition to healthcare services, (b) inclusion of services and costs regardless of the costs being related to the autism and who incurred them, (c) relatively less use of costly inpatient service, maybe because of the children’s age range (Höfer et al., 2022), and (d) the gatekeeper function of the general practitioner in the Dutch healthcare system with coordinating care, stepped-care model, and preventing secondary care use (Zwaanswijk et al., 2011). The total utilization and costs in our study at both time points could be higher compared to other studies because of the first three arguments, while they might have been lower by the latter two arguments. Since these arguments affect the data on both time points, the difference pre- and post-diagnostically should not be affected, besides a potential age effect on the costly use of inpatient services (Croteau et al., 2017; Höfer et al., 2022). However, a higher response rate for the questionnaires on utilization of the health-related services post-diagnostically (at T2) with the hypothesis that more children completed the diagnostics and treatment successfully and, therefore, no longer participated in the study, could have enlarged the decrease in total annual and healthcare costs, and increased the child’s QoL as well.

Using primary caregivers to collect health-related service utilization information has clear advantages, such as the accessibility and availability of the data, and consistency of the same caregiver reporting over time. Importantly, not all health-related utilization data in our study could have been found in files or otherwise accessible sources, such as family and other sector costs. Potential disadvantages are the time-consuming task for the respondent, the subjectivity of or bias in the provided information, and potential variability in reports over time (Hakkaart et al., 2024). The most frequent problem in collecting health-related utilization data is underreporting, which may be related to a long recall period, high utilization frequency, and type of utilization, such as outpatient services or mental health services (Bhandari & Wagner, 2006; Zuvekas & Olin, 2009). Especially in the latter type of utilization, experienced stigmatization and shame may lead to underreporting (Bhandari & Wagner, 2006). With the cost estimates based on the utilization data, underestimation of the costs is conceivable. Because the informant of the utilization data was the same on both time points, we assumed some continuity in potential underestimation of utilization and associated costs pre- and post-diagnosis for all the children. This could have a greater impact when compared with other studies. Also, caregivers' estimations might have been biased by their experiences at different times, possibly overestimating costs pre-diagnosis (hectic referral periods) and underestimating post-diagnosis (clearer diagnosis and helpful interventions), potentially partly explaining the statistically non-significant cost decline. As part of the healthcare service utilization, we found more children using prescribed (44 to 53%) and over-the-counter medication (11 to 28%) post-diagnosis, both psychiatric and non-psychiatric. Although the children used hardly any psychoactive medication pre-diagnosis, the increase in the medication costs was partly due to an increase in the use of psychoactive medication post-diagnosis. Most psychoactive medication was used with regard to ADHD symptoms (36% of all children) and behavior problems (17% of all children). Croteau et al. (2017) reported psychoactive medication use to be common in their study and questioned the accessibility to long-term care with a reduction of psychiatric medical visits over time, while we found a stable but more focused health-related service utilization. Note that the contribution of total medication costs in our study was less than 5% of the total costs post-diagnosis, including all sorts of medication. In the Canadian study, Croteau et al. (2017) found a rising contribution of total (psychoactive) medication costs of 5% to 9% (year 1 to year 2) following a diagnosis, maybe since there was already more medication use pre-diagnosis and older ages were included than in our study. In contrast, Höfer only found a contribution of 3% in children to 11 years old with an autism spectrum diagnosis visiting German outpatient clinics, also including a broad scale of services. Reasons for increased use may include targeted intervention post-diagnosis and a wider range of pharmacological treatment options available for older children, as outlined in guidelines (Lord et al., 2022; Volkmar et al., 2014). Increased psychiatric medication use in older children could be due to more behavioral problems (Croteau et al., 2017; Shimabukuro et al., 2008), but our findings did not support this in our age group.

The study design limitations prevent us from accurately estimating causal relationships, particularly regarding service utilization and quality of life. Additionally, as children age, their needs and level of independence evolve, which can independently impact the variables under consideration. To illustrate, Cidav et al. (2013) found decreasing costs because of less physical therapy, occupational therapy, and speech therapy for preschool to school-age children but increasing costs due to more outpatient and inpatient care, psychiatric medication, personal guidance, and respite services. They reported an increase of 5% in costs for each year of age, with top increases up to the age of 16. In our study, we certainly could not rule out an age effect explaining the service utilization and cost fall over the diagnosis boundary. The sample size did not allow us to stratify for age. However, our regression analysis of age on the total costs showed no effect pre- and post-diagnosis. Replication in larger study samples, including adolescents with potentially higher service use and costs (Buescher et al., 2014), is needed. While we observed trends over time, attributing causality requires other study designs.

Exploring determinants of post-diagnostic total costs, we identified the pre-diagnostic total costs and the post-diagnostic quality of life as such. The child’s better quality of life was associated with lower total cost. This finding would seem logical, while resources are probably less needed for children functioning rather well. However, multivariable regression analysis showed pre-diagnostic costs as the only independent factor, explaining 23% of the variance, with lower costs pre-diagnosis related to lower costs post-diagnosis. Although we observed major shifts in service utilization and variability in the individual trajectories, this finding suggests some stability in the total service utilization need over time, regardless of the diagnosis, which requires further research. In contrast to other studies (Rogge & Janssen, 2019), we detected no other child or caregiver factors associated with post-diagnostic total costs, probably due to power problems in our relatively small study sample.

### Strengths and Limitations

Although this study benefits from a longitudinal design with repeated measurements in a well-defined group of autistic children, using validated instruments including the ADOS-2, a broad societal perspective, and bottom-up cost calculation, several important limitations should be considered when interpreting the results.

First, limitations may stem from the small study sample size, with consequences for the statistical analyses. Because of the exploratory nature of this study and the potential importance of these novel findings, we adapted to this limitation using non-parametric tests and a limited number of variables in the multivariable regression analyses. So, non-significant results might be realistic but also be explained by power problems. Because we could not stratify our results by the autism level and age of the children, we performed two univariate regression analyses. Further research with larger sample sizes and a broader age range, including adolescents, is needed to replicate our study findings, also exploring service use and cost beyond our age group (Buescher et al., 2014; Cidav et al., 2013; Croteau et al., 2017).

Second, data on specific interventions in the interval from pre- to post-diagnosis was not available. Some intervention service use might be included in the post-diagnosis measurement, and care as usual is more or less standardized according to international guidelines (Lord et al., 2022; Volkmar et al., 2014). Also, in restricting the estimated service utilization and costs to the children, we did not include the broader societal perspective of the caregiver or family with the risk of underestimation. For example, previous studies with a broad societal perspective found considerable proportions of caregiver productivity loss between nearly ten to over thirty percent of the total costs (Buescher et al., 2014; Rogge & Janssen, 2019). Therefore, including the intervention information, caregiver service utilization, informal care, and productivity loss to capture the complete picture should be the next step in future research.

Third, the generalizability of the study results is limited due to the data-collection in rather well-functioning but clinically referred autistic children of foremost Dutch origin (Table 1) compared to other study samples (Barrett et al., 2012; Cidav et al., 2013); this might have led to potential bias in the reported service use and costs. Hence, the results do not apply to autistic children in the general population. Although we collected data on children in a Dutch multicenter study, the international generalizability of our study results is limited due to the differences in health systems and provided services between countries. Consequently, a broader replication of the study from an international perspective should capture a more complete picture.

Lastly, using the EQ-5D-3L proxy version by the caregiver to assess the health-related QoL of the child may have consequences. Research shows that autistic children often rate their quality of life higher than caregivers (Clark et al., 2014; Sheldrick et al., 2012). The improvement in mean total utility score from 0.58 to 0.66 was not significant in our study, and both scores were significantly lower (p < 0.01) than the age- and sex-adjusted Dutch general population norm score (0.94; Stolk et al., 2009). Also, the mean utility score of 0.66 post-diagnosis was about the same as the mean utility score of 0.67 on the EQ-5D-3L proxy version in a Dutch study with 88 autistic children during the diagnostic phase (Ten Hoopen et al., 2020) and the mean utility score of 0.66 on the HUI3 proxy measure in a US study with 218 children of an autism network (Payakachat et al., 2014). Next to the impact of autism (Kuhlthau et al., 2013), there might be an underestimation of the child’s QoL by the caregivers. Thus, self-reports should be preferred unless children are too young or disabled to report for themselves (Coghill et al., 2009). Finally, although an instrument such as the EQ-5D-3L to measure health-related QoL has some advantages like feasibility, applicability in a broad age range and diverse health states, and the calculation of preference-based utilities for comparison’s sake, the question is whether a generic, not autism-specific instrument is sensitive enough to measure the health-related QoL in autistic children and whether the preference-based utilities of the EQ-5D-3L are helpful for such an exploration, also potentially resulting in underestimation of the differences across the diagnostic boundary.

## Conclusion and Implications

In this exploratory study, we evaluated the quality of life and societal costs of autistic children before and after receiving an autism diagnosis, as reported by the caregivers. Notwithstanding the limitations of our study, such as lacking information about informal care and the caregiver’s productivity as a substantial part of the societal costs associated with autism in children, we observed remarkable trends of decreased annual total costs over time while the child’s quality of life seemed to improve and educational needs appeared to be better met. Healthcare costs fell by 50%, with less somatic and more mental healthcare. The results support the timely clarification of autism traits in children for effective intervention and treatment planning to potentially improve the quality of life and reduce societal costs. This information can help us understand the need for services and appropriately allocate resources, particularly in ongoing policy debates about organizing youth (mental) health services and related youth care domains.

## Supplementary Information

Below is the link to the electronic supplementary material.

Supplementary file1 (DOCX 39 KB)
