# Supplementary material for: Quality of Life and Societal Cost in Autistic Children: An Exploratory Comparative Study Pre- and Post-Diagnosis
Source: J Autism Dev Disord. 2025 Mar 9;56(8):3129–41. doi: 10.1007/s10803-025-06760-9 (PMC13391643; doi:10.1007/s10803-025-06760-9)
Supplement: Supplementary file 1 — Supplementary file1 (DOCX 39 KB) [file 10803_2025_6760_MOESM1_ESM.docx]

Quality of Life and Societal Cost in Autistic Children:

An Exploratory Comparative Study Pre- and Post-Diagnosis

**Supplementary File S1** Overview of health-related cost categories

| Healthcare sector costs | Child and family costs | Other sector costs |
| --- | --- | --- |
| General practitioner  Physiotherapy  Occupational therapy  Speech therapy  Dietitian  Homeopath or acupuncturist  Social worker  Mental health institution  Psychologist, psychiatrist (practice)  Hospital outpatient clinic  Hospital day treatment  Hospital admission  Admission otherwise  Medication use, prescribed  Medication use, over-the-counter | Homework guidance  Special diet | Youth care  Family guardian  Special needs daycare  Remedial teaching at school  Guidance at school  Special needs after-school care  Weekend foster care  Community crisis care  Foster care  Police contacts  Detention  Other services |

**Supplementary File S2** Unit costs for the health-related service use in Euro 2021

| Health-related service | Units | Unit costs (€) |
| --- | --- | --- |
| General practitioner | Consultations | 36.97 |
| Physiotherapist | Consultations | 36.97 |
| Occupational therapist | Consultations | 36.97 |
| Speech therapist | Consultations | 32.86 |
| Dietician | Consultations | 35.00 |
| Homeopath, acupuncturist | Consultations | 55.00 |
| Social worker | Consultations | 70.00 |
| Mental healthcare | Consultations | 124.27 |
| Psychologist, psychiatrist – practice | Consultations | 104.75 |
| Youth care/welfare | Contacts | 78.75 |
| Family guardian | Contacts | 80.00 |
| Special needs daycare | Days | 232.10 |
| Remedial teaching | Contacts | 65.00 |
| Homework guidance - private | Out-of-pocket payment | Costs reported |
| Extra school guidance | Contacts | 22.90 |
| Special needs after-school care | Days | 70.49 |
| Weekend foster care | Days | 363.39 |
| Community crisis care | Days | 242.00 |
| Foster care | Days | 19.55 |
| Police | Contacts | 78.75 |
| Detention | Days | N.A. |
| Other services | Contacts | Supplementary file 3 |
| Hospital outpatient clinic | Consultations | Supplementary file 3 |
| Hospital day admission | Days | 198.10 |
| Hospital admission | Days | 696.31 |
| Admission otherwise | Days | 335.83 |
| Diet | Out-of-pocket payment | Costs reported |

*N.A.* not applicable (service not used)

**Supplementary File S3** Unit costs for the health-related service use otherwise in Euro 2021

| Health-related other services and outpatient clinics otherwise specified | Units | Unit costs (€) |
| --- | --- | --- |
| Unspecified | Contacts | 109.03 |
| Special care for children with hearing and communication problems | Contacts | 32.86 |
| Special treatment and guidance for autistic children and adults | Contacts | 124.27 |
| Magnetizer | Contacts | 50.00 |
| Neurofeedback | Consultations | 62.20 |
| Orthoptist | Contacts | 30.43 |
| Parent training by youth mental health | Contacts | 124.27 |
| Parent training by an academic child & adolescent psychiatric outpatient clinic | Contacts | 124.27 |
| Pedagogic home support | Contacts | 133.51 |
| Personal guidance of child (home support) | Contacts | 64.70 |
| Personal guidance of parents (home support) | Contacts | 64.70 |
| Outpatient clinic for specialized toilet training | Consultations | 100.65 |
| General hospital, outpatient clinic | Consultations | 89.35 |
| Academic medical center, outpatient clinic | Consultations | 180.75 |
| Unspecified outpatient clinic | Consultations | 100.65 |
| Surgical outpatient clinic | Consultations | 81.13 |
| Neurologic outpatient clinic | Consultations | 109.89 |
| Pediatric outpatient clinic | Consultations | 111.94 |
| Emergency room | Consultations | 287.56 |
| Treatment center for children with language & speech problems | Consultations | 32.86 |
| Virologist | Consultations | 100.65 |
| Specialized school guidance | Contacts | 65.00 |

**Supplementary File S4** Unit costs for prescribed medication in Euro 2021

| Prescribed medication | Unit | Unit costs (€) |
| --- | --- | --- |
| Aerius® | Tablet 2,5 mg | 0.24 |
| Amoxicillin® | Daily dose for child | 1.72 |
| Antibiotics unspecified | Daily dose for child | 1.72 |
| Aripiprazole | Tablet 1 mg | 1.65 |
| Aripiprazole | Tablet 5 mg | 0.07 |
| Aripiprazole (Abilify®) | Tablet 10 mg | 2.04 |
| Atrovent® nebulizer | 2 ml | 0.44 |
| Avamys® nasal spray 27.5 mcg. | Dose | 0.06 |
| Bactrimel® | Ampul | 4.94 |
| Clonidine | Tablet 0.025 mg | 0.09 |
| Depakote syrup | Dose | 0.03 |
| Depakote liquid | Daily dose for child | 0.87 |
| Dipiperon® (Pipamperone) liquid 40 mg/ml | Daily dose for child | 0.06 |
| Eczema ointment | Dose | 0.10 |
| Erythrosine liquid | Daily dose for child | 4.21 |
| Flixotide® (Flucatisone) Aerosol | Dose of 0.124 mg | 0.12 |
| Forlax® (Macrogol) junior sachets | Sachet 4 gr | 0.23 |
| Kalium phosphor (Vitamin D6) | Tablet | 0.042 |
| Keppra® | Tablet 1000 mg | 0.45 |
| Klyx ® enema | Enema dose 120 mg | 1.31 |
| Magnesium | Tablet 400 mg | 0.23 |
| Melatonin | Tablet 0.25 mg | 0.020 |
| Melatonin | Tablet 1 mg | 0.44 |
| Melatonin Circadin ® | Tablet 2 mg CR | 0.69 |
| Melatonin | Tablet 3 mg | 0.29 |
| Melatonin | Tablet 5 mg | 0.17 |
| Methylphenidate | Tablet 5 mg | 0.08 |
| Methylphenidate | Tablet 10 mg | 0.09 |
| Methylphenidate (Ritalin®) | Tablet 10 mg | 0.13 |
| Methylphenidate (Medikinet®) | Capsule CR 20 mg | 0.93 |
| Methylphenidate (Medikinet®) | Capsule CR 30 mg | 1.14 |
| Methylphenidate (Concerta®) | Tablet CR 18 mg | 0.87 |
| Methylphenidate (Concerta®) 27 mg. | Tablet CR 27 mg | 1.19 |
| Methylphenidate (Concerta®) 54 mg. | Tablet/capsule CR 54 mg | 1.62 |
| Nexium | Tablet 20 mg | 0.30 |
| Omeprazole | Capsule 20 mg | 0.03 |
| Losec® (Omeprazole) | Capsule 20 mg | 0.59 |
| Penicillin | Daily dose for child | 1.72 |
| Qvar® aerosol 100 mcg | Dose | 0.10 |
| Risperidone | Tablet 1 mg | 0.07 |
| Risperidone oral solution 1 mg/ml | Dose 0.4 mg | 0.08 |
| Salbutamol Aerosol | Dose 100 mcg | 0.013 |
| Seretide® Aerosol 25/50 | Dose | 0.19 |
| Silicea® (Vitamin D12) | Tablet | 0.054 |
| Strattera (Atomoxetine) | Capsule 40 mg | 6.10 |
| Thyrax Duotab® | Tablet 25 mcg | 0.03 |
| Ventolin® Aerosol | Dose 100 mcg | 0.016 |
| Vitamin D | Tablet 20 mcg | 0.06 |

Reference prices via National Health Care Institute ([www.medicijnkosten.nl](http://www.medicijnkosten.nl); [www.farmacotherapeutischkompas.nl](http://www.farmacotherapeutischkompas.nl)).

**Supplementary File S5** Unit costs for over-the-counter medication in Euro 2021

| Over-the-counter medication | Unit | Unit costs (€) |
| --- | --- | --- |
| Bisolvon® | Daily dose for child | 0.54 |
| Coughing syrup | Daily dose for child | 0.54 |
| Melatonin | Tablet 10 mg | 0.32 |
| Melatonin | Tablet 0.20 mg | 0.25 |
| Melatonin | Tablet 0.10 mg | 0.026 |
| Nasonex® nasal spray | Daily dose two-sided | 0.08 |
| Nasal spray unspecified | Daily dose two-sided | 0.22 |
| Nasal spray xylometazoline | Daily dose two-sided | 0.12 |
| Orthiflor Original® | Capsule | 0.29 |
| Paracetamol supp. | Suppository | 0.30 |
| Paracetamol (Panadol®) | Tablet 500 mg | 0.09 |
| Paracetamol | Tablet 500 mg | 0.03 |
| Paracetamol supp. | Suppository 200 mg | 0.23 |
| Sleepzz® | Tablet 5 mg | 0.47 |
| Trafloxal® | Daily dose one-sided | 0.27 |

Reference prices via National Health Care Institute ([www.medicijnkosten.nl](http://www.medicijnkosten.nl); [www.farmacotherapeutischkompas.nl](http://www.farmacotherapeutischkompas.nl))

or average prices from consumer websites

**Supplementary File S6** Univariate single variable regression analyses of all independent child, caregiver, and family variables with total annual costs at t2 (post-diagnosis) of autistic children

| Variables | Total annual costs at t2 | | | | |
| --- | --- | --- | --- | --- | --- |
|  | *B* | *SE* | *t* | *p* | 95% CI |
| *Child variables* |  |  |  |  |  |
| Age | -1272.406 | 797.270 | -1.596 | 0.120 | [-2892.653;347.841] |
| Functioning (CBCL) | -70.509 | 258.724 | -0.271 | 0.788 | [-596.437;456.320] |
| Autism (ADOS-2) | -253.275 | 1055.478 | -0.240 | 0.812 | [-2398.264;1891.715] |
| Quality of life at t1 (EQ-5D) | -12822.704 | 7618.254 | -1.683 | 0.102 | [-28304.858;2659.451] |
| Quality of life at t2 (EQ-5D) | -23515.853 | 10264.534 | -2.291 | **0.028*** | [-44375.896;-2655.809] |
| Total annual costs at t0 | 0.463 | 0.137 | 3.374 | **0.002**** | [0.184;0.741] |
|  |  |  |  |  |  |
| *Caregiver variables* |  |  |  |  |  |
| Age caregiver | -190.686 | 416.955 | -0.457 | 0.650 | [-1038.988;657.616] |
| Functioning caregiver (ASR) | 18.746 | 207.923 | 0.090 | 0.929 | [-404.276;441.769] |
| Parenting stress (OBVL) | 41.642 | 162.828 | 0.256 | 0.800 | [-289.635;372.918] |
| Family functioning (FAD) | -164.864 | 485.440 | -0.340 | 0.736 | [-1154.927;825.198] |
|  |  |  |  |  |  |

* p ≤ 0.05; ** p ≤ 0.01

*CI* confidence interval, *T0* Pre-diagnostic phase, *T1* Diagnostic phase*, T2* Post-diagnostic phase*, CBCL* Child Behavior Checklist, *ADOS-2* Autism Diagnostic Observation Schedule-2, *EQ-5D* EuroQoL Five Domain Health Questionnaire, *ASR* Adult Self-Report, *OBVL* Opvoedingsbelasting vragenlijst [Parenting Stress Questionnaire], *FAD* Family Assessment Device.
